# Comparative study of microscale and macroscale technique for encapsulation of *Calotropis gigantea* extract in metal-conjugated nanomatrices for invasive ductal carcinoma

**DOI:** 10.1038/s41598-023-39330-z

**Published:** 2023-08-18

**Authors:** Ayesha Aftab, Bashir Ahmad, Shazia Bashir, Saima Rafique, Muhammad Bashir, Tayyaba Ghani, Asma Gul, Atta Ullah Shah, Ranjha Khan, Abdulrahim A. Sajini

**Affiliations:** 1 Department of Biological Sciences, International Islamic University, H10 Campus, Islamabad, Pakistan; 2 Department of Physics and Applied Mathematics, Pakistan Institute of Engineering and Applied Sciences, Islamabad, Pakistan; 3 Department of Physics, Air University, Islamabad, Pakistan; 4 Department of Metallurgy and Material Engineering, PIEAS, Islamabad, 45650 Pakistan; 5 National Institute of Laser and Optronics (NILOP), Islamabad, 44000 Pakistan; 6 UCSF/Benioff Children’s Hospital, San Francisco, CA USA; 7 Department of Biomedical Engineering, Khalifa University of Science and Technology, Abu Dhabi, 127788 United Arab Emirates; 8 Healthcare Engineering Innovation Center (HEIC), Department of Biomedical Engineering, Khalifa University, Abu Dhabi, 127788 United Arab Emirates

**Keywords:** Biotechnology, Nanoscience and technology

## Abstract

The encapsulation of plant extract in nanomatrices has limitations due to its adhesion to walls, size control, high cost and long durations that results in low yield. Macroscale and microscale level techniques for development of micro/nanoparticles may impact the encapsulation of plant extract. This study aimed to evaluate the relative efficiency of microscale and macroscale techniques for encapsulation of plant extract, which is not compared yet. Keeping this in view, encapsulation of *Calotropis gigantea* leaves extract (CaG) was attained in silver-conjugated poliglusam nanomatrices (POL/Ag) to induce apoptosis in invasive ductal carcinoma (IDC) cells. The ethanolic CaG extract was prepared using percolation method and characterized by chemical tests for its active phytochemical compounds. The droplet-based microfluidic system was utilized as microscale encapsulation technique for CaG in nanomatrices at two different aqueous to oil flow rate ratios 1.0:1.5, and 1.0:3.0. Moreover, conventional batch system was utilized as macroscale encapsulation technique consisted of hot plate magnetic stirrer. The prepared nanomatrices were analysed for antioxidant activity using DPPH test and for cytotoxicity analysis using MCF-7 cells. The characteristic peaks of UV–Vis, FTIR and XRD spectrum confirmed the synthesis of CaG(POL/Ag) by both the encapsulation methods. However, microfluidic system was found to be more expedient because of attaining small and uniform sized silver nanoparticles (92 ± 19 nm) at high flow rate and achieving high encapsulation efficiency (80.25%) as compared to the conventional batch method (52.5%). CaG(POL/Ag) nanomatrices found to have significant antioxidant activity (*p* = 0.0014) against DPPH radical scavenging activity. The CaG(POL/Ag) of the smallest sized formulated by the microfluidic system has also shown the highest cytotoxicity (90%) as compared to batch method (70%) at 80 µg/mL. Our results indicate that the microscale technique using microfluidic system is a more efficient method to formulate size-controlled CaG(POL/Ag) nanomatrices and achieve high encapsulation of plant extract. Additionally, CaG(Pol/Ag) was found to be an efficient new combination for inducing potent (*p* < 0.0001) apoptosis in IDC cells. Therefore, CaG(Pol/Ag) can be further tested as an anti-cancer agent for in-vivo experiments.

## Introduction

Nanoparticles have been studied extensively in cancer research because of their unique characteristics which facilitates scientists in diagnostic and treatment of cancer^1^. These particles can be formulated in distinctive ways to control particle size to not only act as anti-cancer agents but also serve as delivery vector for drug. As compared to conventional drugs, nanodrugs emerges as more significant improving stability, biocompatibility, biodegradability, crossing blood–brain barriers and especially providing a valuable targeted therapy^1,2^. In cancer treatment nano drug delivery is providing a more promising efficacy because they can be formulated to provide a shell (e.g. liposome), capsules (oil/water or polymeric membrane), embedding matrix like nanomatrices (polymeric) or pH-temperature sensitive drug-conjugation^3^. The conjugation of polymer embedding metallic nanoparticles is a hybrid combination and may provide an embedding and conjugation system to achieve high-definition encapsulation of any anti-cancer agent^3,4^.

Invasive ductal carcinoma (IDC) is one of the most common globally occurring and lethal type of breast cancer^5^. The recent research is focused to prevent and treat this cancer at early stages^6,7^. However, the synthetic chemo-drugs available in the market but comes with severe side effects. Medicinal plants are source of natural products with various biological activities like antiviral, hypoglycaemic, antibiotic, antioxidant, anticancer, antifungal, anti-hypertensive, and insecticide^8^. *Calotropis gigantea* (*C. gigantea*) is one of the abundantly occurring medicinal plant that usually found in northern areas of Pakistan^9,10^, and is comprised of many bioactive components including phenols, flavonoids, alkaloids etc.^11,12^. The rich source of phytochemicals makes it possible to use *C. gigantea* against several illness like inflammatory, microbial infection, wound healing,^13^ and apoptosis of cancer cells^9,10,14^. Furthermore, the extract of plant like CaG is a mixture of multiple bioactive components and their delivery at cancer site is very difficult in human complex body^15,16^. To overcome the problem of biodistribution and bioavailability of degradable plant extracts the advanced research has focused on preparation of nano or micromatrices^17^. These encapsulating matrices will not only safely deliver the plant extract but will also enhanced their therapeutic efficacy^18^. The extract of *C. gigantea* (CaG) has not been previously formulated with any nanoparticles for IDC cells and it is reported in this study.

The encapsulation of plant extract in micro/nanoparticles such as polymers, cyclodextrins, solid dispersions and liposomes has emerged to be a most competent encapsulating agents^18^. Poliglusam is extensively used as microcarrier/micromatrices for safe delivery of anti-cancer and anti-microbial drugs as well as the plant-based drugs because of its natural biodegradable groups and performance delivery^17,19,20^. The poliglusam micromatrices can also be conjugated to the metals like silver nanoparticles (AgNPs) which will not only aid the encapsulation efficiency of poliglusam but also increase the cytotoxicity of nanoparticles^21^. These arrangements and modifications provide enhance surface interaction with cancer cells and allow extract to combat resistance in complex human body^22–24^.

There are several methods, reported previously, for encapsulation of plant extract at micro and macroscale level^25,26^. The encapsulation methods can be divided into two techniques on basis of size of reactor for mixing reagents^26,27^. The microscale level consists of reactor that is 1 to 100 μm in diameter^28,29^. While reactor above 100 μm forms the macroscale level for encapsulation^27^. Microscale techniques may include the microemulsions^30^ formed by microfluidic system and phase inversion precipitation^29^. The macroscale techniques may include the spray drying, hot plate magnetic stirring, fluid bed coating, and ion gelation methods of encapsulation^25^. These methods and type of nanoparticles used in encapsulation depends upon the chemical nature of plant extract, i.e. oil based, volatile or high water soluble^16,25^. However, they have many different reported limitations such as extract adhere to the walls in spray drying, takes long duration for encapsulation, high cost due to use of high amount of nanoparticles and size control of encapsulation material^25^. These problems will not only cause denaturation of medicinal plant extract but will also results in low yield of encapsulation in nanomatrices. Microfluidic system is an efficient and precise technique for synthesis of size and shape control nanoparticles including nano/micromatrices. The PMMA microchip-based encapsulation of plant extract has been performed previously with up to 77.125% encapsulation efficiency (EE) of micromatrices^31^. The PMMA microchip was used for water in oil microemulsion in order to synthesize controlled sized nanoparticles and encapsulate the plant extract^31^. These microchips can also be modified for oil in water emulsion system depending upon the nature of plant extract.

To the best of our knowledge, the relative significance of microscale and macroscale techniques for encapsulation of plant extract has not been reported so far. Therefore, this research focuses on studying and comparing the two techniques at microscale and macroscale level for loading plant extract in nanomatrices. For this purpose, the CaG plant extract has been encapsulated in poliglusam-silver (Pol/Ag) nanomatrices at macroscale using conventional batch method and microscale using microfluidic system. We hypothesised that microfluidic system is a competent method for encapsulation of plant extract as compared to conventional wet chemical method (conventional batch method). This new combinational nanomatrices were also examined for its antioxidant and anti-proliferative effect against IDC cells.

## Material and methods

### Material

Poliglusam (low molecular weight, CAS: 9012-76-4, Sigma, gifted from University of Science and Technology of China), silver nitrate (AgNO_3_, Duksan), ethanol, acetic acid, canola oil (Sigma), sodium hydroxide (NaOH), 2,2-diphenyl-1-picrylhydrazyl (DPPH, Sigma), dimethyl sulfoxide (DMSO), chloroform, sulphuric acid, hydrogen chloride (HCl), Mayer’s reagent, ammonia solution and ferric chloride. All mentioned chemicals are of analytical grade.

### Plant collection and extract preparation

The plant materials used in this study were collected from wild areas in Islamabad, Zero-point Latitude: 33° 41′ 37.00" N and longitude: 73° 03′ 54.00" E, after identification by the Dr. Mushtaq Ahmad, Director of Botanical Garden & Herbarium of Islamabad, Pakistan and Quaid-i-Azam University Islamabad, Pakistan. The plant specimen was authenticated by comparison with herbarium specimen of Islamabad Herbarium of Pakistan and submitted with voucher number ISL-769 in this herbarium for future reference. Following the rules of national action plan of the Pakistan, the leaves of plant (*Calotropis gigantea* (L.) W.T. Aiton, http://legacy.tropicos.org/Name/2603210?projectid=32) were cut with clean scissors and brought for further identification by renowned plant taxonomist (Quaid-i-Azam University Islamabad). The research was in compliance with local policies and regulations of Federal region. The plant was washed with distilled water, dried under shade and ethanolic extract was prepared by following the reported percolation method^31^ at Pakistan Institute of Engineering and Applied Sciences (PIEAS), Islamabad, Pakistan. The percentage yield of extract was calculated using the following formula.

$$\% yield=\left(\frac{We}{Ws}\right)X 100$$

Here, *W*_*e*_ is the weight of dried extract and *W*_*s*_ is the weight of dried CaG leaves sample. The CaG leaves were air dried and weigh, while extract solvent was evaporated using rotatory evaporator (SENCO) at 30 °C and weighed for percentage yield calculation^31^.

### Preliminary phytochemical screening of CaG extract

The phytochemical analysis of ethanolic CaG extract was accessed by different chemical tests for terpenoids, flavonoids, phenols, and alkaloids by the reported tests^12,32,33^. For terpenoids detection, chloroform was added to plant extract in ratio of 2:5 and then 1 mL of sulphuric acid was added. For alkaloids, equal amount of HCL and extract was added. After mixing the three drops of Mayer’s reagent were added. The presence of flavonoids was indicated by the yellow colour when ammonia solution was added followed by few drops of sulphuric acid into the extract in equal ratio. Lastly, for phenols detection the ethanolic extract was diluted with distilled water and three drops of 10% ferric chloride was added^33^.

### Experimental setups for synthesis and encapsulation

**Figure 1:**
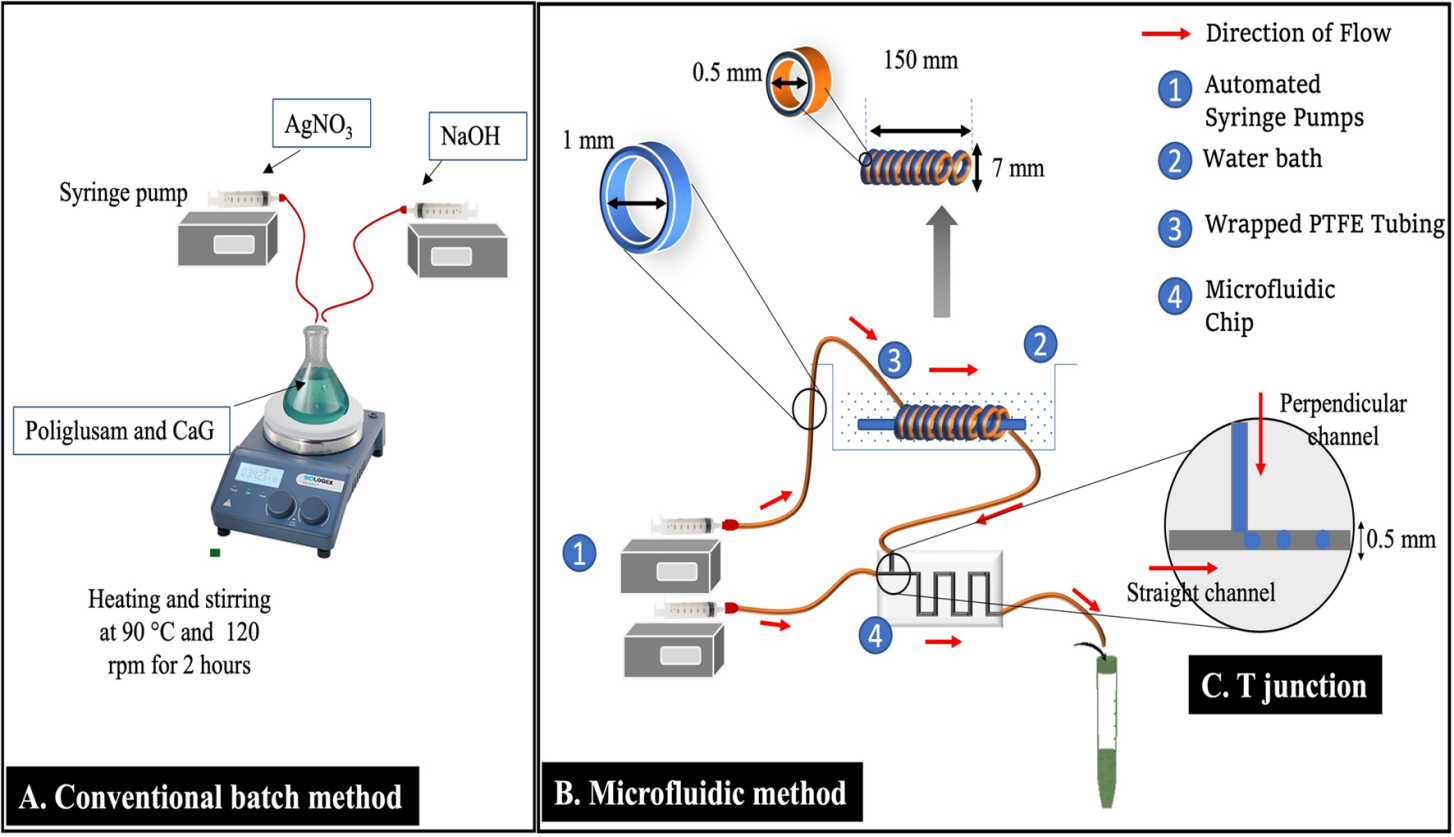
The experimental setup for synthesis of CaG-loaded POL/Ag. (**A**) Conventional batch method using magnetic stirrer. (**B**) Automated microfluidic system consisted of syringe pumps and microchip of 0.5 mm channel. (**C**) T junction and the perpendicular and straight channels for formation of microemulsion in microchip.

The Pol/Ag was prepared by the conventional batch method and microfluidic systems in order to compare the encapsulation of CaG using the macroscale and microscale techniques, respectively. The experimental setup is illustrated in Fig. 1.

### Macroscale encapsulation using conventional batch setup

The experimental setup of conventional batch method consisted of hot plate magnetic stirrer to prepare the CaG loaded poliglusam-silver nanomatrices (CaG-(Pol/Ag)) at macroscale^34^ with few modifications as shown in Fig. 1A. The 1.5% of poliglusam (prepared in 2% of acetic acid solution) was subjected to heating and magnetic stirring at 95 °C and 120 rpm, respectively. A 4 mM of AgNO_3_ solution was added drop wise into poliglusam solution in ratio 2:1 (poliglusam : AgNO_3_) and left for 2 h of stirring. For encapsulation of CaG, 10 mg/mL of CaG was mixed into the poliglusam solution and AgNO_3_ was added drop by drop. After collection of nanomatrices the 10% of NaOH was added drop wise in volumetric ratio of 0.1:1; NaOH: AgNO_3_. These samples were washed three times with deionized water (d.H_2_O) by centrifugation at 10,000 rpm for 30 min each time and lyophilized for storage at room temperature and characterization.

### Microscale encapsulation using microfluidic system

The microfluidic system was used for synthesis of CaG-(Pol/Ag) at microscale which is shown in Fig. 1B. Two syringe pumps were used for dispensing of precise flow rates which are controlled by automated computer system. The poly-methyl methacrylate (PMMA) microchip developed previously in our lab^35^ having T junction was used for the microemulsion formation. The straight channel carries the continuous phase (canola oil) and perpendicular channel carries the dispersed phase (poliglusam, CaG and AgNO_3_ reaction mixture) shown in Fig. 1C. The dispersed phase contains 1.5% of poliglusam, 4 mM of AgNO_3_ and 10 mg/mL of CaG. First of all the dispersed phase is passed through the polytetrafluoroethylene (PTFE) microreactor immersed in water bath to heat the solution up to 90 °C^31^. The homogenous and stable microemulsions (water-in-oil) were formed by controlling the flow rates of both the phases. The total flow rate (TFR, mL/h) ratio of aqueous: oil was set as 0.4: 0.6 (F1) and 1.8: 5.4 (F2) and relative flow rate (RFR) ratio of 1:1.5 (F1), and 1:3 (F2). The samples were collected and 10% of NaOH was added to them (0.1:1, NaOH: AgNO_3_) for acceleration of reduction of AgNO_3_. The collected samples were washed and stored. In order to compare both methods with equal parameters, the stirring time set for batch system was 2 h which equals to the time taken by microfluidic system to collect equal volume of nanomaterial suspension.

### Determination of encapsulation efficiency

The percentage encapsulation efficiency (%EE) of Pol/Ag nanomatrices synthesized by microfluidic system and conventional batch method was calculated by measuring the free drug present in samples. For this, the CaG extract from Pol/Ag nanomaterial was separated using 10,000 rpm centrifugation for 30 min and the amount of free drug in supernatant was calculated using UV–Vis spectrophotometer at 340 nm^31^. The %EE was calculated by following formula.

$$\%EE=\frac{Tp-Fp}{Tp}X 100$$

where *Tp* is total CaG used in encapsulation and *Fp* is free CaG detached from Pol/Ag.

### Characterization of nanomaterial

The physical characteristics, morphology and size of particles were characterized by scanning electron microscope (SEM), X-ray powder diffraction (XRD), Fourier-transform infrared spectroscopy (FTIR), Dynamic light scattering (DLS) and ultraviolet visible (UV–Vis) spectroscopy.

### Total antioxidant capacity (TAC)

The TAC of CaG and CaG-loaded nanomaterial was evaluated by 1, 1-Diphenyl-2- picrylhydrazyl (DPPH) solution test by using the reported protocol^32^. 0.1 mM of DPPH solution was prepared in ethanol and immediately absorbance was taken at 517 nm for control reading using 96 well plate spectrophotometer (Thermo Fisher scientific). The different concentrations, 40, 60, 80, 100, 120 and 160 μg/mL of ascorbic acid, CaG, and CaG-(Pol/Ag) nanomatrices were prepared in DMSO, and DPPH solution was added in ratio of 1:3; DPPH: antioxidant. The mixtures were shaken vigorously and placed in dark for 30 min at room temperature. The absorbance of each sample was measured at 517 nm. Here, the ascorbic acid was used as standard.

### Anti-cancer activity

The CaG-(Pol/Ag) nanomatrices produced from batch and microfluidic methods were tested against the IDC cells (MCF-7) by MTT assay^31^. The 96 well plate was prepared by seeding MCF-7 (1X10^7^ cells/well) cells and cultured in RPMI media for 24 h at 37 °C. The different formulations of nanomaterial with different concentrations were added into the wells and further incubated for 24 h. Afterwards, the media was removed and MTT dye (5 mg/ mL) was added into each cell, incubated for 2 h and DMSO was added to solubilize the formazan crystals. The absorbance of each cell was taken at 570 nm using the micro-plate reader. the percentage (%) cell viability was calculated using the following formula.

$$\% Cell\,viability=\frac{Abs\left(sample\right)}{Abs\left(control\right)}X 100$$

where, the abs(sample) and abs (control) are the absorbance of sample and control respectively.

### Statistical data analysis

The statistical data analysis was performed using GraphPad prism v.9. IC50 values were calculated and Dunnett’s multiple comparisons test was applied. Two-way ANOVA was also accessed. The threshold values were set as 0.05 and sstatistical significance was established when *p < 0.05, **p < 0.01, ***p < 0.001 and ****p < 0.0001. The nanomaterial and droplet size were measured by ImageJ software. The Mean and standard deviation (SD) were calculated by Graph Pad prism v.9

## Results

### Plant extract phytochemical analysis

The CaG extract was prepared using the ethanol as solvent by percolation method. The 50 g of dried leaves of CaG has given 9.8 g of ethanolic extract and the yield of 19.6%. This extract was found to be positive for terpenoids, alkaloids, flavonoids and phenols as indicated by reddish-brown layer at interface, orange, yellow and green colour respectively. Those findings are in accordance with previous reported studies^9,10,12^ In another research, the ethanolic extract of *C. gigantea* has 46.75 mg of flavonoid and 33.71 mg of phenolic content^14^. The previous reported studies from HPLC and GCMS data also confirms the presence of several pharmacological antioxidant compounds^36–38^ like terpenoids like Lupeol^38^, Urs-12-EN-28-Oic acid, oleanolic acid, 3-O-Acetyl-6-Methoxy-Cycloartenol., ketone like; 3.Alpha.-(Trimethylsiloxy) Cholest-5-Ene., sapogenin, natural steroids like; methylcholesta-4,6-Dien-3-One, terpenes like; caryophyllene, farnesene, and several flavonoids^37^ and cardenolides^39^.

### UV–Vis analysis

**Figure 2:**
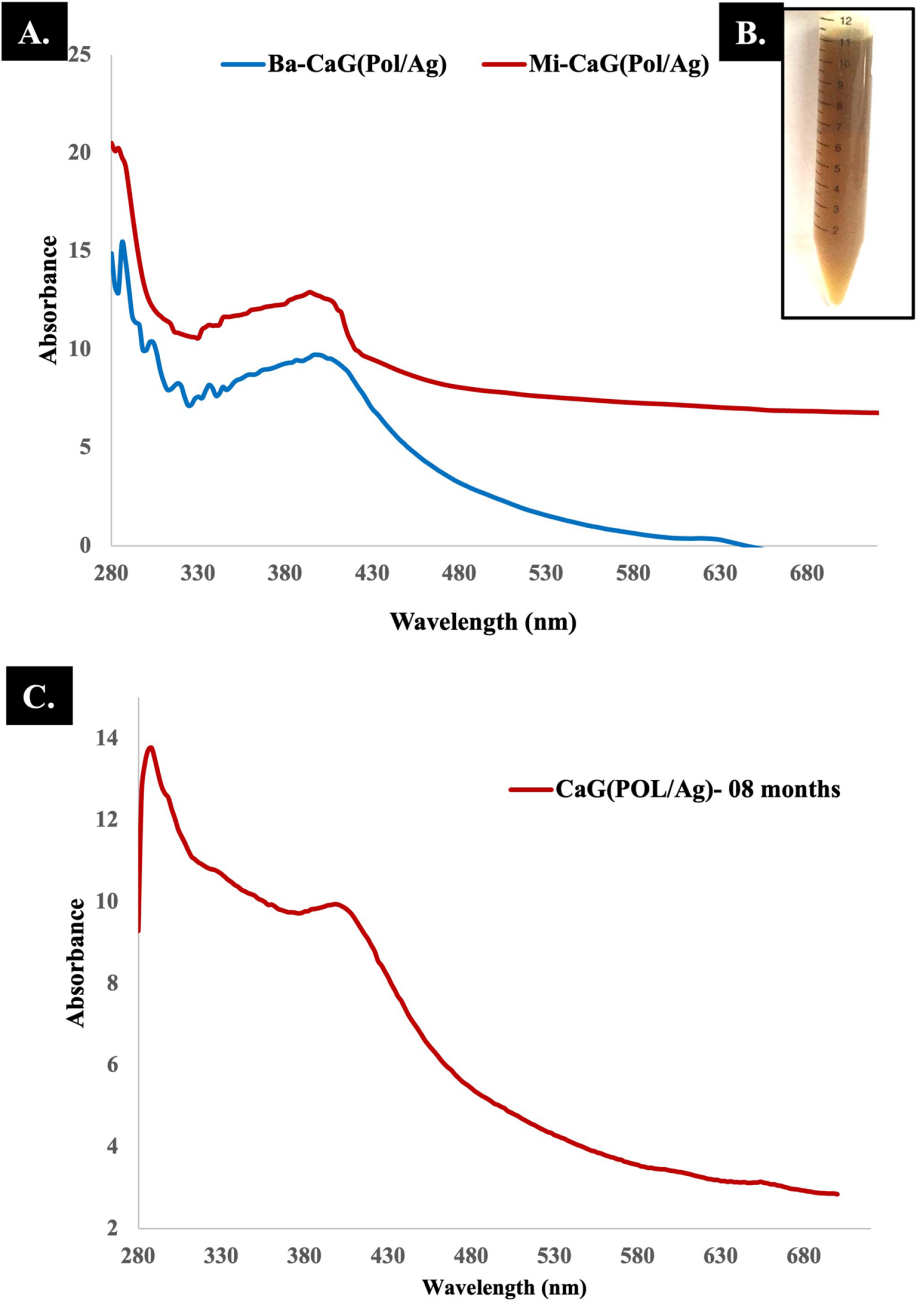
(**A**) The UV–Vis spectra of Ba-CaG(Pol/Ag) and Mi-CaG(Pol/Ag) nanomatrices synthesized by batch and microfluidic system, respectively. (**B**) The suspension of Pol/Ag nanomaterial. (**C**) The UV–Vis spectra of Mi-CaG(Pol/Ag) for eight months stability analysis.

The UV–Vis spectrophotometry of synthesized nanomaterial was performed to confirm the formation of CaG-(Pol/Ag) nanomatrices. The change in color from clear to reddish brown indicated the formation of Pol/Ag nanomatrices as shown in Fig. 2B^31,35^. The UV–Vis characteristic peaks of Pol/Ag nanomatrices synthesized by conventional batch method (Ba-CaG(Pol/Ag)) and microfluidic system (Mi-CaG(Pol/Ag)) are shown in Fig. 2A, where an absorbance is visible near 400 nm because of surface plasmon resonance (SPR) of silver nanoparticles^40^. The silver nanoparticles synthesized by microfluidic system has relatively more absorbance with narrow UV–Vis spectrum, which justify the narrow size distribution of nanoparticles synthesized by microfluidic system. The CaG has peak at 340 nm which is representing the effective encapsulation of CaG in nanomaterial. Furthermore, the peak around 280 nm shows the presence of poliglusam^20^. Altogether these peaks verify the formation of nanomaterial consisted of CaG loaded in poliglusam and silver nanoparticles^31^. The UV–vis spectrophotometry of CaG(Pol/Ag) prepared via the microfluidic system was performed after eight months to check the stability of the nanomatrices. A peak around 400 nm was observed which confirms the presence and stability of AgNPs as shown in Fig. 2C.

### % EE of composites

The nanomatrices synthesized by microfluidic system were found to have % EE of 80.25%, while batch system has 52.5%. This shows that microfluidic system is relatively a more competent method to successfully encapsulate the high percentage of CaG extract in nanomaterial. This might be because of rapid mixing in microemulsions in the microfluidic chip^31,35,41^. Recently, Aftab et al., has achieved the 77.125 ± 6.9% EE of *Calotropis procera* in chitosan silver nanoparticles using the microfluidic system^31^, while we have not found any other similar study. However, other encapsulation techniques of micro and macro encapsulation has also been reported with many limitations^16,25^ as discussed in Sect. 1.

### FTIR spectrum

**Figure 3:**
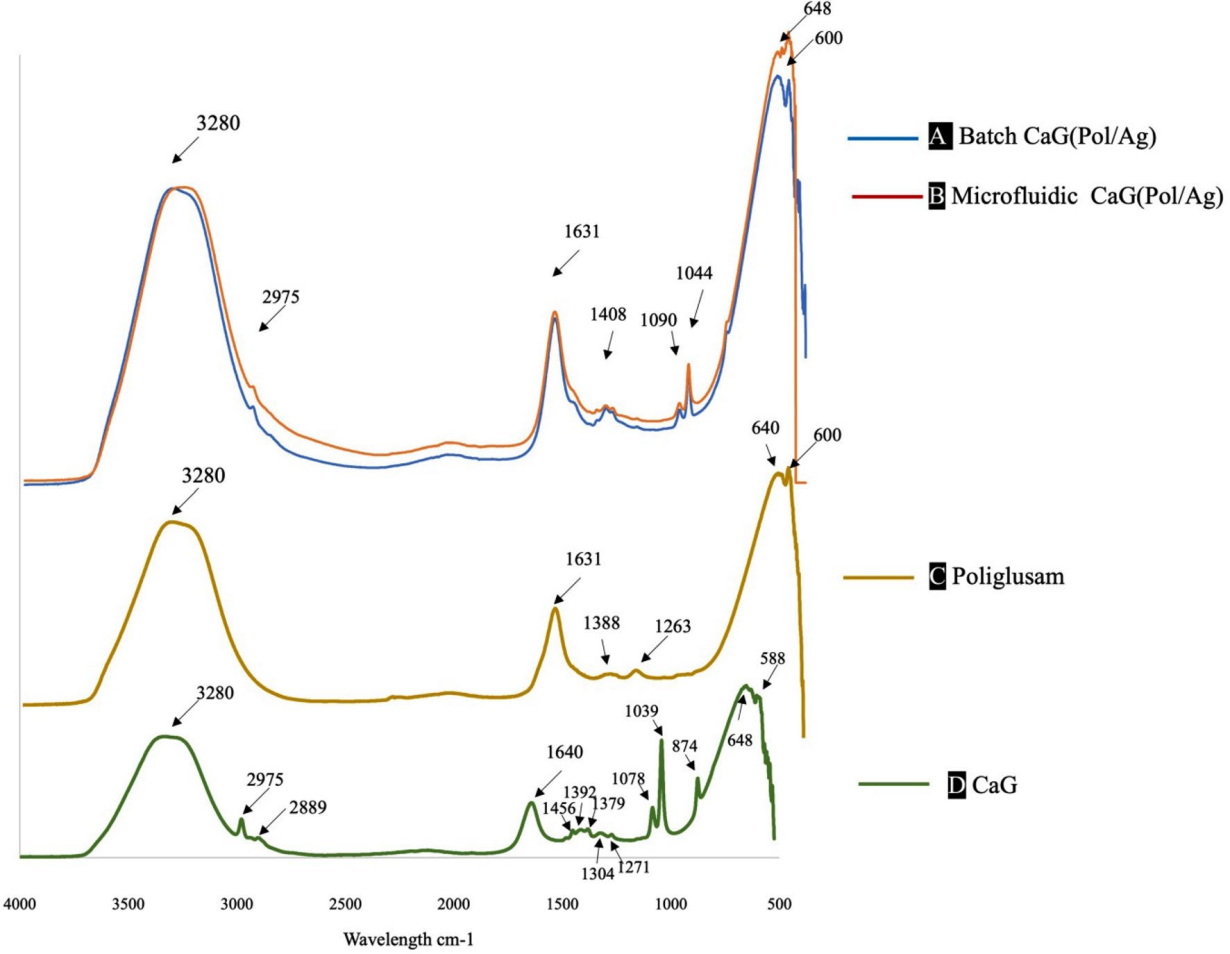
The FTIR of (**A**) Pol/Ag nanomatrices synthesized by conventional batch method and (**B**) microfluidic system, (**C**) poliglusam, and (**D**) *Calotropis gigantea* (CaG).

The absorbance of IR by various functional groups of poliglusam, CaG and Pol/Ag prepared by both methods is shown in Fig. 3. It can be noted that CaG(Pol/Ag) has been successfully prepared by both methods. The shifting of pure poliglusam peaks in Pol/Ag nanomatrices may be because of formation of nanomatrices^31,42^. The broadening and elevation of peak around 3280 cm^-1^ shown in Fig. 3A,B as compared to Fig. 3C, and disappearance of poliglusam peak at 1639.78 cm^-1^ is indicating the role of hydroxyl and amino groups in stabilization of silver ions^23,34^. The CaG has displayed (Fig. 3D) the absorption peaks at various intensity which represents the presence of different functional groups existing in it. The absorbance at 1304 cm^-1^, 1277 cm^-1^ and 1085 cm^-1^ confirming the presence of primary and secondary alcohols, flavonoids, and tertiary alcohol, respectively^43^. These peaks justify the bioactive compounds detected in a forementioned qualitative phytochemical analysis, which plays an important role as antioxidants.

### XRD analysis

**Figure 4:**
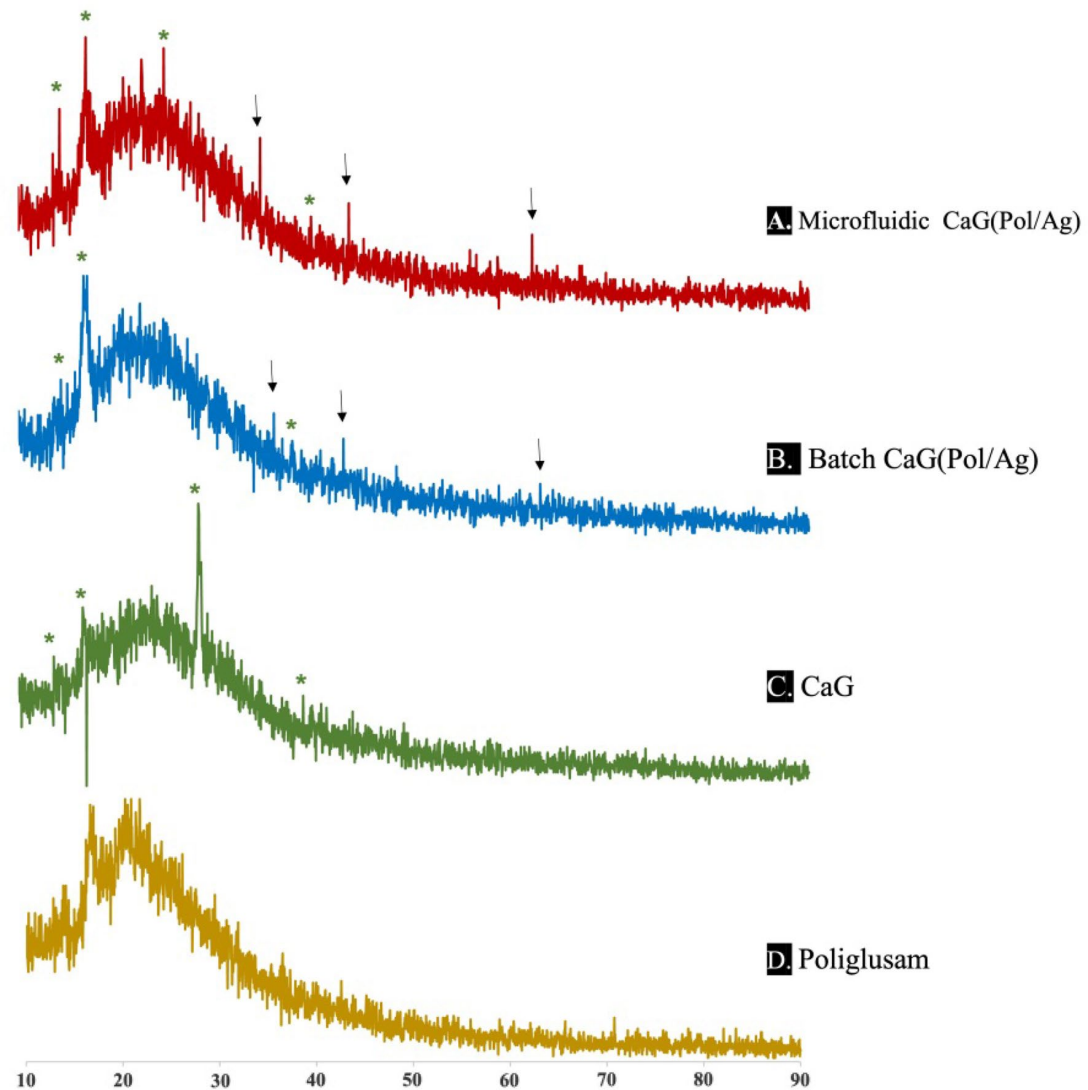
XRD results of CaG(POL/Ag) nanomatrices synthesize through (**A**) microfluidic and (**B**) batch method. (**C**) XRD of CaG extract and (**D**) Poliglusam solution. Arrows indicating characteristic peaks of silver nanoparticles. Asterisks * representing the various peaks of CaG extract.

The 2 θ values of Pol/Ag and CaG analysed by XRD are shown in Fig. 4. The XRD of pure poliglusam (Fig. 4D) has broad peak around 2θ = 20°, which shows the amorphous nature of poliglusam^42^. The POL/Ag prepared by microfluidic system and batch method has broad peak around 20°, along with other peaks which indicates the presence of poliglusam and natural product. For microfluidic system the appearance of Brag reflections at 2 θ = 34.48°, 43.44° and 62° and for batch method at 2θ = 35.84°, 42.88° and 62.8° are clearly indicating the synthesis of silver nanoparticles^42,44^. The nanomatrices (Fig. 4C,D) have a small diffraction as compared to free natural product (Fig. 4B), which is showing the crystalline form of natural product present in the composites^45^.

### SEM analysis for size and structural morphology

**Figure 5:**
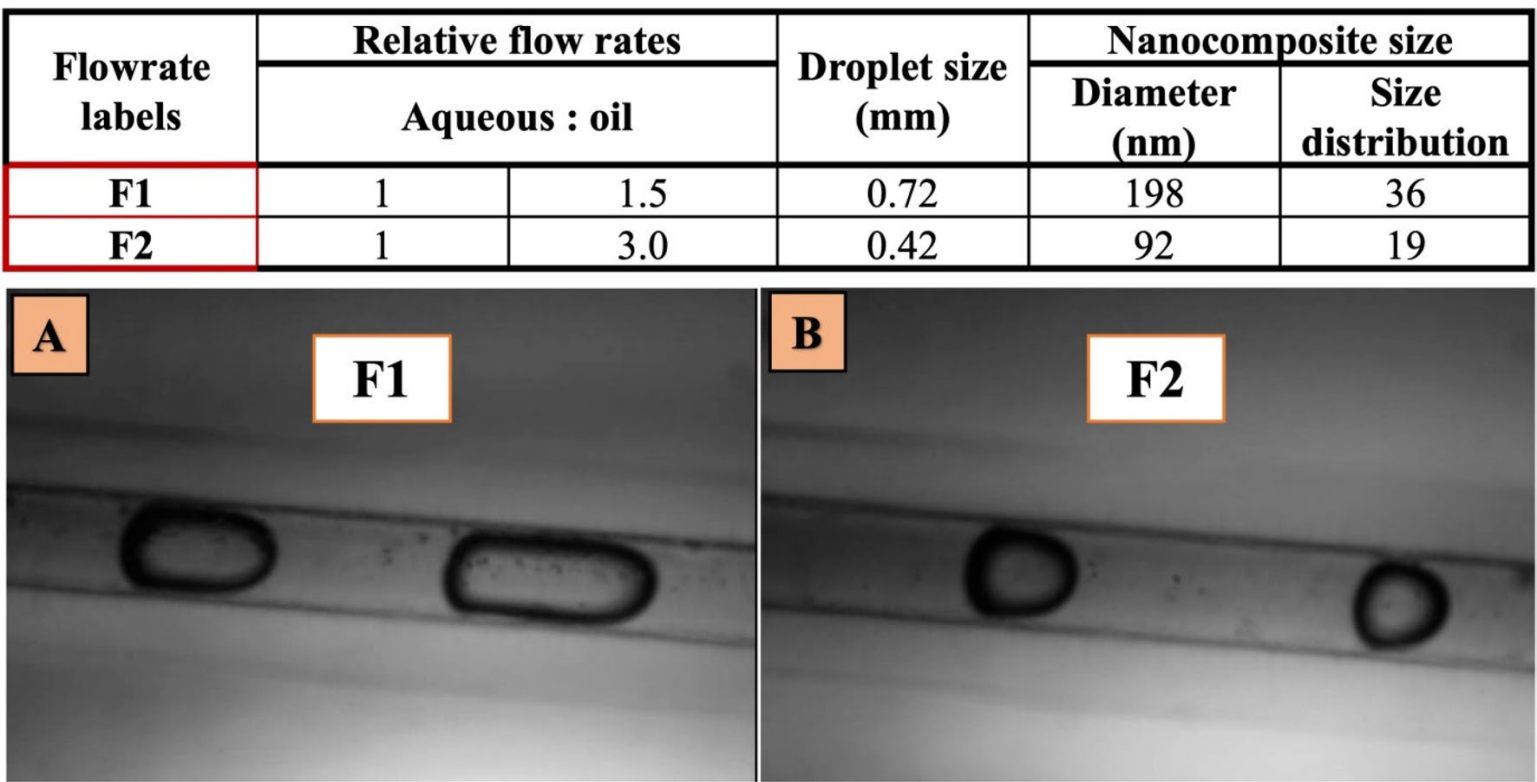
Effect of two different flow rates on droplets and nanomaterial size/size distribution shown in table above. The microchannel and droplets with (**A**) F1 flow rate, and (**B**) F2 flow rate.

**Figure 6:**
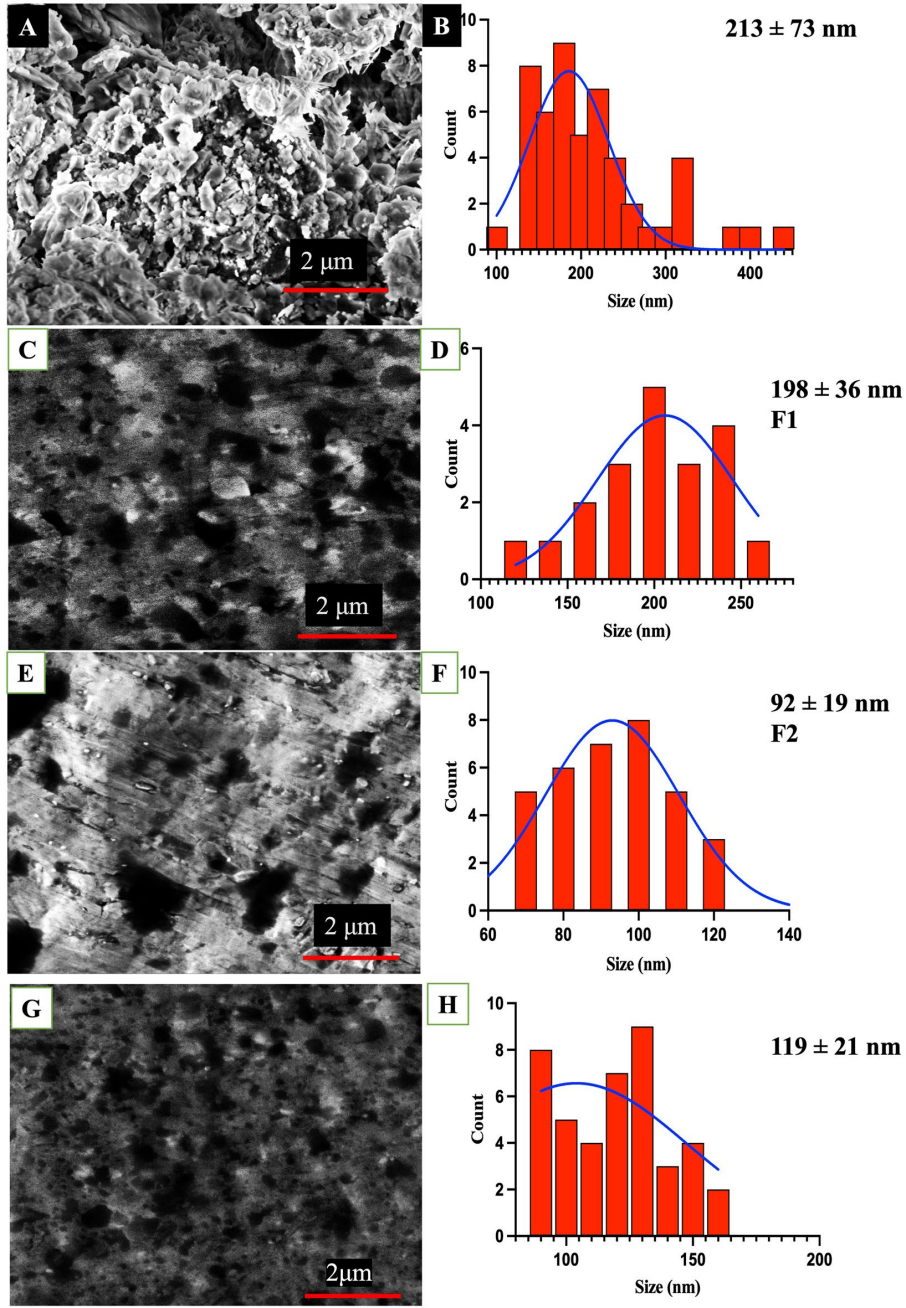
SEM images of CaG(Pol/Ag) nanomatrices. CaG(Pol/Ag) synthesized by batch method is shown in image (**A**) and histogram in (**B**) Synthesised by microfluidic system at different flow rates are shown in images (**C**) and (**E**) and their respective histograms in (**D**) and (**F**). Here flow rate (mL/h) in ratio of aqueous: oil phase is RFR that is F1 = 1:1.5, and F2 = 1: 3. G and H is the SEM image of F2 CaG(Pol/Ag) eight months aged.

The microfluidic system was utilised to tune the nanomatrices size at two different flow rates i.e. F1 and F2 as shown in Fig. 5. The SEM was performed to visualise the nanomatrices for size and morphology shown in Fig. 6A–H.

#### Size analysis

It can be noticed that with the increase in flow rates (TFR and RFR) the size of microemulsion as well the size of silver nanoparticles declined. This is because with the decrease in size of microemulsion the number of nuclei decreases that are formed within each microemulsion which result in decrease in average final size of the nanomaterial^46,47^. The size of microemulsions at two different flow rates in 0.5 mm microchannel was also calculated and has been displayed in Fig. 5. At high flow rate F2, the CaG(Pol/Ag) nanomatrices of size 92 ± 19 nm were formed, while the size of nanomatrices was larger (198 ± 38 nm) at slow RFR (F1). We have also observed a narrow size distribution of nanomatrices with the increase in flow rate. All these finding is in consistent with the previous reports^46–49^. Furthermore, the Pol/Ag nanomatrices prepared by the batch method are relatively large in size and are not evenly distributed (Fig. 6A,B).

#### Morphology analysis

The change in morphology of the nanomaterial captured by SEM was also observed. The SEM images has also shown the bright silver nanoparticles embedded on membrane-type- poliglusam collected from microfluidic system^42^. At high flow rate, the Pol/Ag nanomatrices are more spherical and uniform. On the other hand, the Pol/Ag nanomatrices prepared via a batch method are more irregular in shape/surface. The irregular surface of poliglusam is also not displaying the silver nanoparticles clearly.

#### Stability analysis

The SEM image was taken after eight months to check the stability of nonmaterices. As shown in Fig. 6G,H, after eight months the size of CaG(Pol/Ag) at flow rate F2 recorded was 119 ± 21 nm. The 27 nm increase in size was recorded which is quite negligible and also the CaG(Pol/Ag) were found to be evenly distributed. These results suggest the stability of nanomatrices.

Overall, these finding shows that microfluidic system plays an important in homogenous mixing for synthesis of narrow size distribution and smooth surface of poliglusam microcarriers. These results also indicate that the size and morphology of nanomaterial can be tuned by microfluidic system.^47,49^. Yanar et al. has produced the size control liposomes^48^. While in this research we have formulated the size controlled poliglusam nanomatrices embedding silver nanoparticles conjugated with plant extract, which were not reported earlier.

### Hydrodynamic size and zeta potential

**Figure 7:**
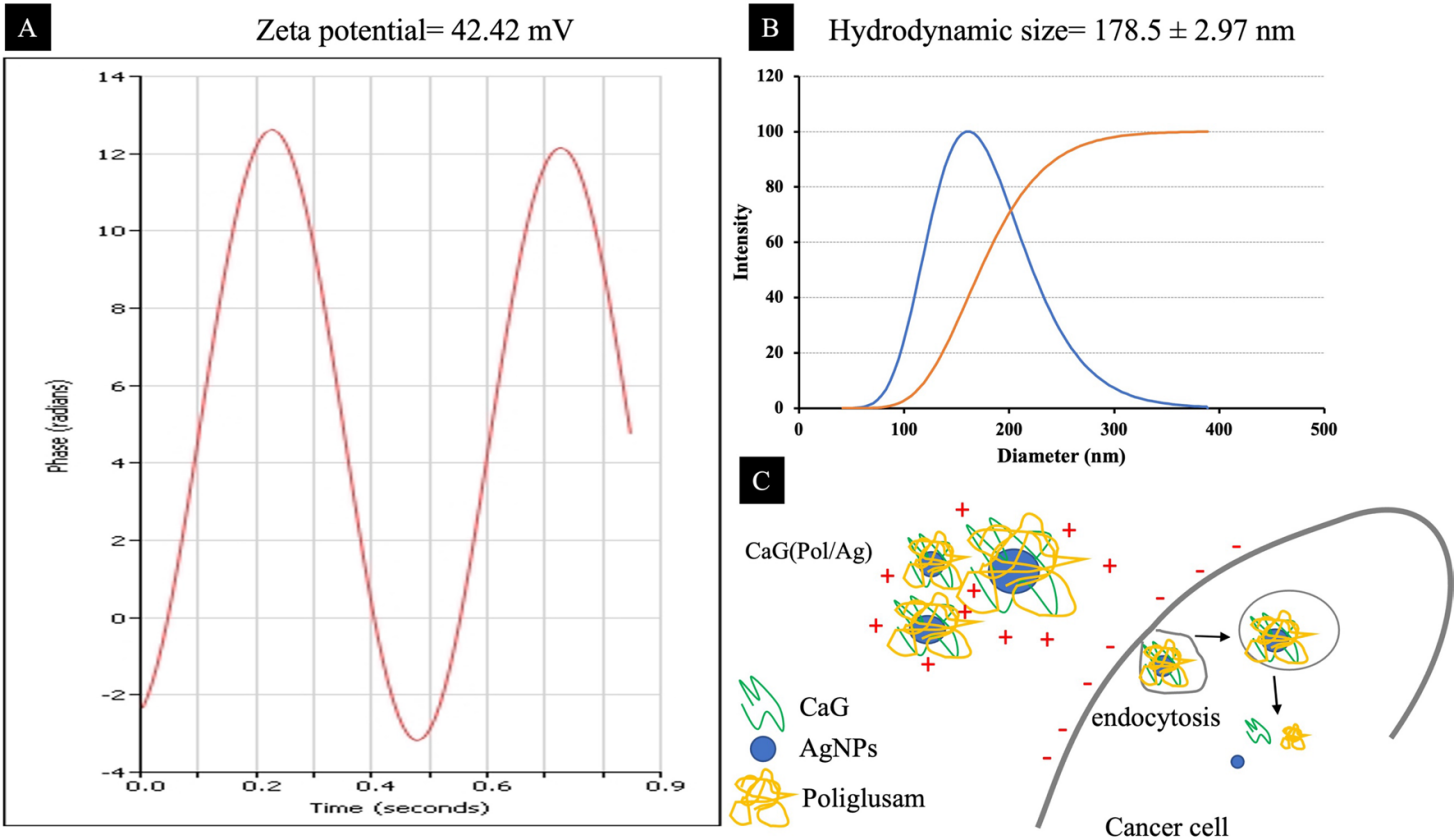
The DLS analysis. (**A**) Zeta potential of CaG(Pol/Ag) nanomatrices. (**B**) Hydrodynamic size calculated via cumulative intensity distribution. (**C**) The diagrammatic representation of electrostatic interaction between CaG(Pol/Ag) nanomatrices and cancer cells aiding the endocytosis.

The hydrodynamic size and zeta potential of the microfluidic prepared CaG(Pol/Ag) nanomatrices was analysed using the DLS technique show in Fig. 7A–C. The zeta potential of any nanomaterial above + 30 mV is considered as highly stable and strongly cationic^50^. in this study the zeta potential of the nanomatrices was found to be 42.42 mV. Which suggests that they are highly stable and cationic. This strong cationic nature of the CaG(Pol/Ag) nanomatrices increase the endocytosis activity on the surface of negatively charged cancer cells due to electrostatic force (Fig. 7C). Therefore, this can be assumed that these nanomatrices would have ability to induce apoptosis in cancer cells to great extent. These results are consistent with earlier reported studies where the electric potential of the particles has been measured to be positively charged^51^. The average size of the nanomatrices was measured to be 178.5 nm with polydispersity index of 2.97. The size measured by the DLS is quite close to the size measured by the SEM analysis. These results indicate that CaG(Pol/Ag) nanomatrices are evenly distributed with positive charge on their surface which make them an ideal nanoagent to induce cancer cell death.

### Antioxidant properties of CaG and nanomatrices

**Figure 8:**
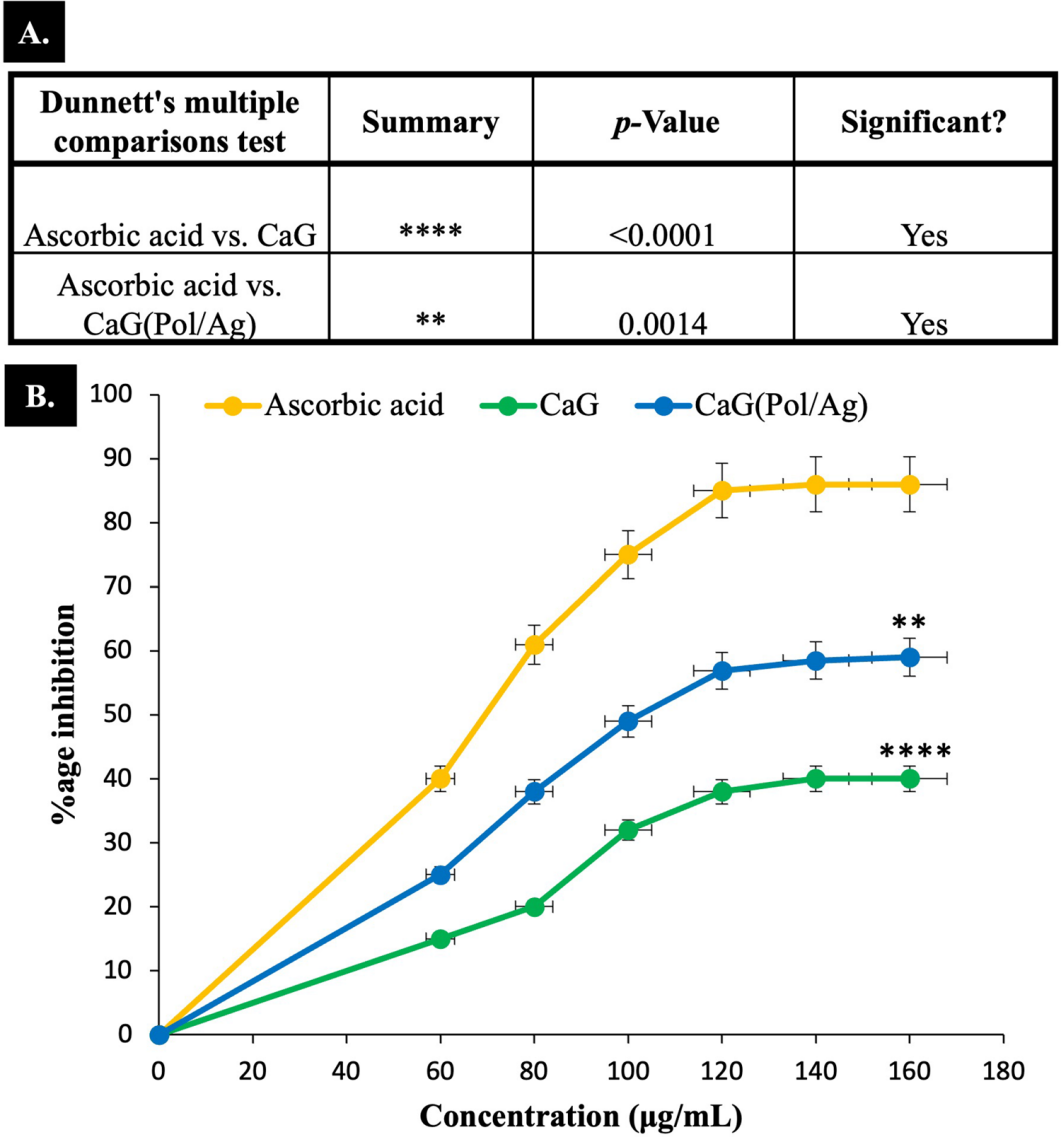
Antioxidant analysis. (**A**) The table statistically demonstrate the significance of CaG(Pol/Ag) by Dunnett’s multiple tests. (**B**).The % inhibition graph of ascorbic acid, calotropis gigantea (CaG) and CaG loaded poliglusam-silver nanomatrices (CaG(Pol/Ag)). Here, n = 3 and **p < 0.01 and ****p < 0.0001.

The reported studies have found valuable phytochemical present in CaG including alkaloid, flavonoids, and phenols. Therefore, it can be used as a natural antioxidant^9,10,12^. The percentage inhibition of free radicals by CaG, CaG(POL/Ag) and standard is shown in Fig. 8. The results shows that CaG alone has adequate potential of antioxidant activity. However, when combined with Pol/Ag nanomatrices, the CaG(Pol/Ag) possess a significant antioxidant activity.

### Anti- cancer effect

**Figure 9:**
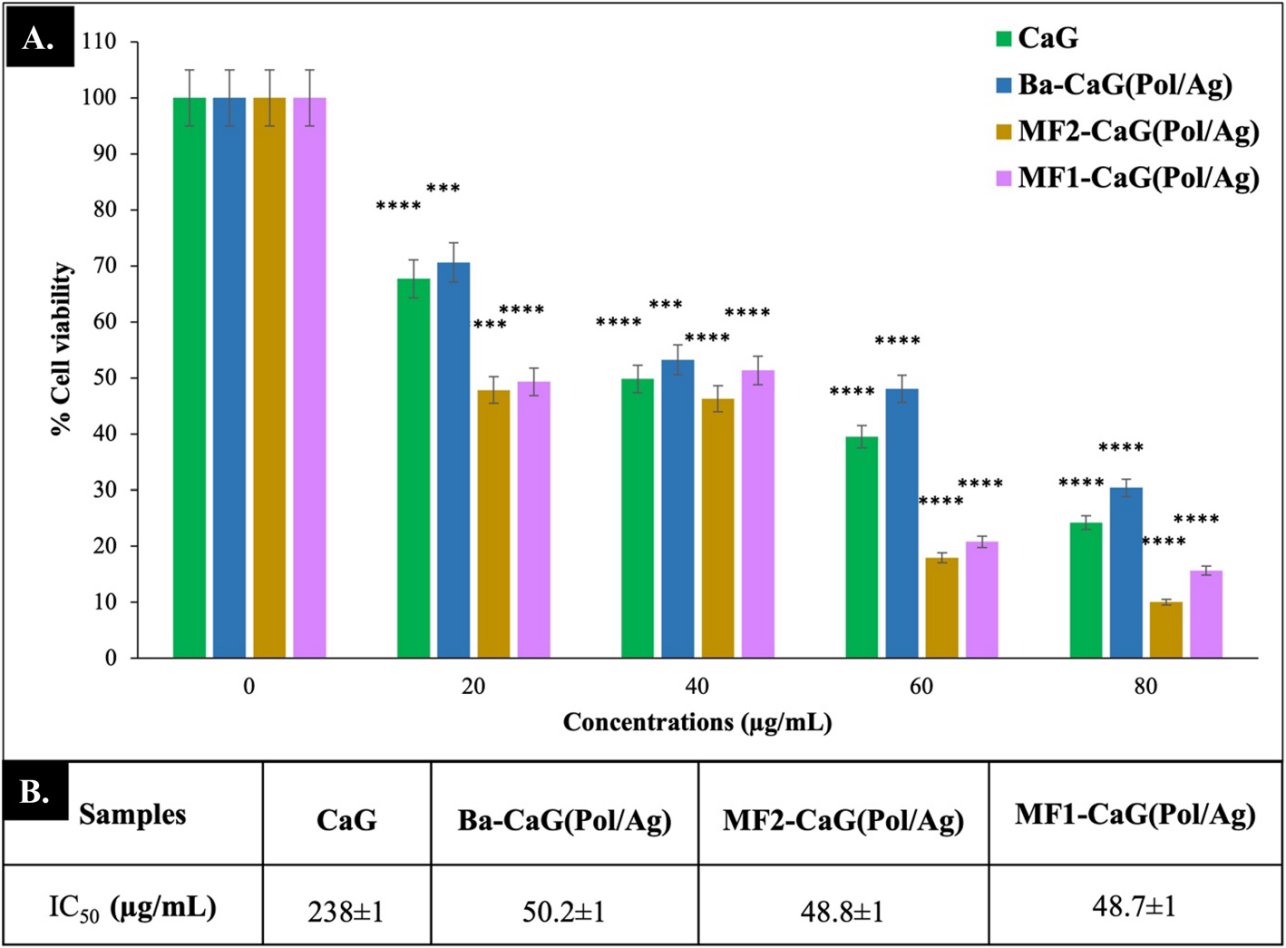
(**A**) The % cell viability of CaG extract and CaG-Pol/Ag nanomatrices synthesised by batch method (Ba-CaG(Pol/Ag)) and microfluidic system method, MF1-CaG(Pol/Ag) of 198 ± 36 nm and MF2-CaG(Pol/Ag) of 92 ± 19 nm. (**B**) The IC50 values of each agent against MCF cells.

In this study, the IDC cells, MCF-7, were treated with the microchip-formulated (M-CaG(Pol/Ag)) nanomatrices of two different sizes, MF1-CaG(Pol/Ag) of 198 ± 36 nm and MF2-CaG(Pol/Ag) of 92 ± 19 nm, and batch method synthesized (Ba-CaG(Pol/Ag)) nanomatrices (213 ± 73 nm). Their cytotoxic effect and IC_50_ values are shown in Fig. 9A,B respectively. The CaG(Pol/Ag) nanomatrices synthesised by two different methods has shown quite significant (*p* < 0.0001) anti-cancer activity in increase concentration dependant manner. However, M-CaG(Pol/Ag) nanomatrices has shown more decrease in IDC cells viability as compared to Ba-CaG(Pol/Ag) nanomatrices. At 60 and 80 µg/mL of dose a difference of 30% and 20% of cell viability can be noticed in Fig. ﻿9 by MF2-CaG(Pol/Ag).

Furthermore, we have also found that smallest size particles 92 ± 19 nm (MF2-CaG(Pol/Ag) at 80 µg/mL has the minimum cell viability of 10% (% cytotoxicity = 90). While Ba-CaG(Pol/Ag) nanomatrices has lowest cell viability of 30% (% cytotoxicity = 70 ) at 80 µg/mL. These results indicate that with the change in size and morphology of nanoparticles, the cell viability of the cancer cells is also affecting. A small size and uniform morphology of Pol/Ag nanomaterial has more potent anticancer efficacy^52–54^. However, in previous studies scientist has also preferred the 50 nm size over 10 or 20 nm^55,56^, this may be because the smallest particles may escape the cancer cells easily even before performing their average activity. While, in our study, the size of particle is around 92 nm, which is quite optimal to enter and escape the cancer cells upon their average activity^55^.

It can also be observed in this study that the antioxidant-CaG extract has shown 24% cell viability at 80 µg/mL, however conjugating it with tuned size and morphology Pol/Ag nanomaterial has increased the anti-tumour activity^31^. The two-way ANOVA test has confirmed those finding to be statistically significant, *p* < 0.0001.

## Conclusion

The present study reports that encapsulation of plant extract at microscale using microfluidic system is more efficient to set out high yield encapsulation efficiency as compared to encapsulation at macroscale using conventional batch method. Also, the microfluidic system provides the flexibility to control the size, shape, and polydispersity of nanomaterial by changing the flow rates of the agents. This research reports the CaG(Pol/Ag) as a new nanotherapeutic agent which is competent to induce apoptosis in MCF-2 cells.

## Data Availability

All data generated or analyzed during this study are included in this published article. The Plant *c. gigantea* was collected by following the rules of national action plan of the Pakistan and was dully verified and permitted by Herbarium of Pakistan (Islamabad) Quaid i Azam university Islamabad, Pakistan and identified by renowned plant taxonomist of Islamabad (*Calotropis gigantea* (L.) W.T. Aiton, http://legacy.tropicos.org/Name/2603210?projectid=32.
